# Co-infection of *Nocardia* and *Aspergillus fumigatus* in a immunosuppressed patient: Case report

**DOI:** 10.1097/MD.0000000000037073

**Published:** 2024-01-26

**Authors:** Lei Wang, Yang Liu, Hui Li

**Affiliations:** a Emergency department, Affiliated Hospital of Shandong University of Traditional Chinese Medicine, Jinan, China.

**Keywords:** *Aspergillus fumigatus*, case report, immunosuppressed, *Nocardia*

## Abstract

**Background:**

*Nocardia* and *Aspergillus fumigatus* are opportunistic pathogenic fungus that has a major impact on the mortality of rheumatoid arthritis patients. Opportunistic infections in immunocompromised patients present diagnostic challenges. *Nocardia* and *A fumigatus* are both easily overlooked because of their rarity, leading to delayed diagnosis and treatment.

**Case presentation:**

We report an infection caused by steroid use in a patient with rheumatoid arthritis. A 76-year-old man with a history of rheumatoid arthritis was admitted to our hospital because of cough, expectoration and fever for 10 days. The patient had low immune function, granulocytopenia, diffuse infiltration could be seen on chest computed tomography, and BAL fluid galactomannan level of 1.3 S/CO. The microbiological findings reflect a possible co-infection with *Nocardia* and *A fumigatus*. Voriconazole was used to treat pulmonary aspergillosis, ceftriaxone and Trimethoprim-Sulfamethoxazole were used to treat *Nocardia*. After timely targeted medication administration, the patient was discharged with a good prognosis.

**Conclusion:**

Co-infection is more common in immunosuppressed patients and warrants attention in clinical practice. Early diagnosis and treatment can help patients with Co-infection of *Nocardia* and *A fumigatus* achieve better prognosis.

## 1. Introduction

Immunodeficiency is a state of compromised immune mechanisms that leads to increased vulnerability to opportunistic infections.^[1]^ Opportunistic infections in immunocompromised patients can present a diagnostic challenge. *Nocardia* and *Aspergillus fumigatus* are opportunistic pathogens and easily overlooked owing to their rarity, leading to delayed diagnosis and treatment. In patients with connective tissue diseases, steroids use may lead to an increased risk of opportunistic infections with a poor prognosis. Pneumonia in immunocompromised individuals often has high incidence and mortality rates. However, co-infection with *Nocardia* and *A fumigatus* has rarely been reported. We report a case of pneumonia with *Nocardia* and *A fumigatus* successfully treated.

## 2. Case report

A 76-year-old man with a history of rheumatoid arthritis was admitted to our hospital because of cough, expectoration and fever for 10 days on September 20,2022. Before the patient took methylprednisolone intermittently. On September 10, 2022, the patient developed fever, body temperature up to 38.2ºC, accompanied by cough, yellow and black sputum, occasionally with blood in sputum, chest tightness and shortness of breath. The patient was administered cefalexin by intravenous drip at the local health center. He did not improve and was transferred to our hospital 10 days later.

**Figure 1.**
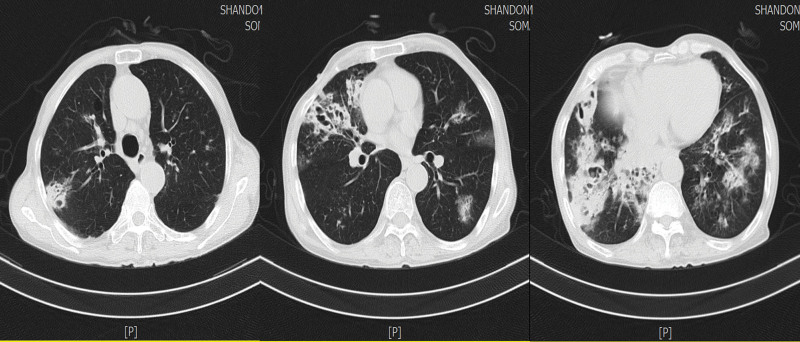
Chest CT performed on September 20, 2022 showing diffuse infiltration and bronchiectasis in both lungs, suggesting infectious lesion. CT = computed tomography.

On admission, he presented with: fever, cough, yellow sputum, fatigue, body temperature of 38.5ºC, blood pressure of 152/91 mm Hg, respiratory rate of 23 breaths per minute, heart rate of 82 beats per minute, normal mental status, emaciation, thick breath sounds in both lungs, and wet rales in both lower lungs. Relevant examinations were as follows: arterial partial pressure of oxygen,71.6 mm Hg; oxygenation index, 217 mm Hg; white blood cell count of 4.28 × 10^9^/L, neutrophil count of 1.8 × 10^9^/L, lymphocyte count of 0.42 × 10^9^/L, CD3 + lymphocyte count of 362.77/μL, CD19 + lymphocyte count of 70.52/μL, hs-CRP level of 162.00 mg/L, creatinine level of 38 µmol/L, albumin level of 20.3 g/L, prealbumin level of 45 g/L, procalcitonin level of 0.59 ng/mL, 1,3-β-d-glucan level of 193.63 pg/mL, galactomannan level of 1.2 S/CO. Liver and kidney function indicators were within the normal limits. Serological tests for HIV, CMV, Epstein-Barr virus, hepatitis C virus, COVID-19, parainfluenza virus, respiratory syncytial virus, influenza A virus, adenovirus, *Chlamydia pneumoniae, Mycoplasma pneumoniae, Legionella pneumophila*, and influenza B virus were negative. Chest computed tomography (CT) showed bronchiectasis and infection in both lungs (Fig. 1).

After admission, the patient was started on intravenous drip cefoperazone sodium/sulbactam sodium (3 g every 8 hours) combined with levofloxacin injection (0.5 g every day) to fight against infection Considering that the patient had bronchiectasis with infection, acetylcysteine atomization was used for expectoration. Bronchoscopic alveolar lavage was performed on the second day after admission, and bronchoalveolar lavage fluid (BALF) was collected for bacterial and fungal cultures and acid fast bacilli detection. BAL fluid galactomannan level of 1.3 S/CO. On the fourth day after admission, the acid fast staining of BLAF fluid was positive, showing 90º branched hyphae, similar to *Nocardia*, and BALF fluid showed gram-positive rods identified later on as *Nocardia* astrosus. The patient BLAF fluid culture was positive for *A fumigatus*. We replaced cefoperazone sodium/sulbactam sodium with ceftriaxone by intravenous drip (2 g every day), and administered Trimethoprim-Sulfamethoxazole (SMZ-TMP) by oral (960 mg every 6 hours) and voriconazole by intravenous drip (400 mg every 12 hours on the first day then 200 mg every 12 hours) for anti infection. The patient did not have fever again, and still had cough and expectoration on the 8th day. However, on the 17th day of treatment, the patient did not have fever again and still had cough, and the chest CT scan showed that the infection focus was absorbed more than before; inflammatory indicators: white blood cell count of 8.98 × 10^9^/L, hs-CRP level of 3.9 mg/L, PCT 0.06 ng/mL, and body temperature were normal. The patient was discharged after 3 days. The patient continued to take SMZ-TMP and voriconazole, and was followed up regularly in the outpatient clinic.

**Figure 2.**
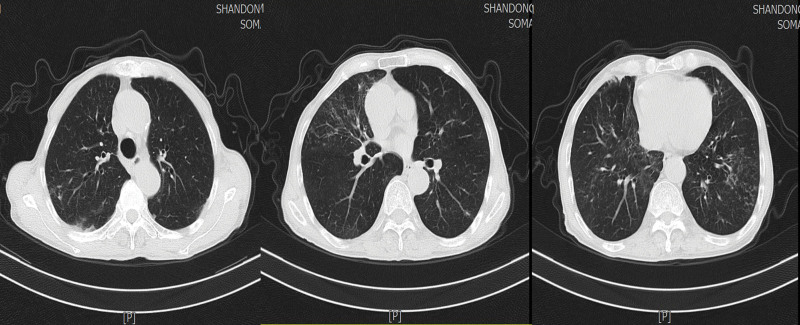
Chest CT performed on March 30, 2023 showing that the lung lesions had improved markedly. CT = computed tomography.

Three months later, the infection index was normal, liver and kidney function was normal, and lung CT showed infection focus was absorbed obviously (Fig. 2). The patient recovered after 3 months of treatment. The patient was very satisfied with the treatment.

## 3. Discussion

In this case, the patient had low immune function, granulocytopenia, diffuse infiltration could be seen on chest CT, and BAL fluid galactomannan level of 1.3 S/CO. The patient tested positive for *A fumigatus* in the fungal culture. BALF culture revealed *Nocardia*. After treatment with *A fumigatus* and *Nocardia*, the patient condition improved. Therefore, we believe that the patient in the present case was diagnosed with a combination of *A fumigatus* and *Nocardia* infection.

*Nocardia* is a Gram-positive rod-shaped aerobic mycobacterium belonging to the order *Actinomycetales*. It has weak acid tolerance and is widely present in soil, air, dust, freshwater, seawater, and decaying plants.^[2]^ *Nocardia* can cause severe morbidity and mortality, particularly in patients with comorbidities or compromised immunity.^[3]^ *Nocardia* infections are typically observed in individuals with compromised immune systems or chronic lung diseases.^[4]^ The main risk factors are defects in T-cell-mediated immunity (e.g., after transplantation), prolonged glucocorticoid therapy, malignancy, graft-versus-host disease, diabetes mellitus, chronic granulomatous disease, and alveolar proteinosis.^[5]^

*Nocardia* often causes pulmonary infections in immunosuppressed hosts.^[6]^ Disseminated nocardiosis is more frequent in immunocompromised patients than in immunocompetent hosts.^[4,7]^The diagnosis of *Nocardia* infection relies on culture based techniques, typically utilizing BALF or sputum samples. Subsequently, molecular techniques were employed for species identification.^[8]^ These isolates are usually susceptible to: TMP/SMZ, carbapenems, amikacin, ceftriaxone, ciprofloxacin, and tazobactam piperacillin. TMP/SMZ is generally selected as the initial therapy, either alone or as the backbone of a multidrug regimen (generally in combination with amikacin, imipenem or third-generation cephalosporin).^[9]^

*Nocardia* is an opportunistic pathogen that most frequently affects the lungs. A retrospective study was conducted based on medical records of consecutive adult patients (N = 60) with nocardiosis hospitalized during 2007 to 2018 at a tertiary hospital in central Israel. Multivariable conditional logistic regression models were fitted. Immunosuppressive pharmacotherapy was positively associated with nocardiosis (matched odds ratio [OR] 4.40, 95% confidence interval [CI] 2.25–8.62, *P* < .001), particularly corticosteroid therapy (matched OR 4.69, 95% CI 2.45–8.99, *P* < .001). Systemic corticosteroid therapy was strongly associated with pulmonary nocardiosis (matched OR 5.90, 95% CI 2.75–12.66, *P* < .001).^[10]^ A retrospective observational analysis of clinical and microbiological data collected from 55 cases of *Nocardia* pneumonia between 2010 and 2016 in 5 Spanish hospitals. Thirty-four patients were receiving systemic and/or inhaled corticosteroids prior to infection, 8 had neoplasia, and 6 had hematological malignancies.^[8]^ Nocardiosis a severe infection, with a miscellaneous clinical spectrum and complex treatments.

Aspergillus species are ubiquitous molds primarily found in the environment such as soil and decaying vegetation. The genus contains more than 200 species, in which 30 species are reported to cause human infections.^[11]^ *A fumigatus* is an environmental mold and opportunistic pathogen that causes invasive aspergillosis (IA), a life threatening pulmonary infection that affects patients with immune defects that disrupt the barrier functions of the lung.^[12]^ Most people are exposed to Aspergillus spores and breathe them daily without getting sick; however, individuals with compromised immune systems or lung diseases have a higher risk of developing active infection.^[13]^ Voriconazole remains the preferred treatment, and isazole and posaconazole have similar therapeutic effects with less toxicity. Combination therapy can be used to treat severe infections and immunosuppression.

Patients on immunosuppression are at risk of unusual infections. Risk factors for Aspergillus infection include prolonged corticosteroid usage, T-lymphocyte immunosuppressants, neutropaenia and hematopoietic cell transplant recipients; it can be localized to the lungs or may disseminate to other organs.^[14]^ A number of studies have long shown that high-doses and prolonged treatment courses of glucocorticoids correlate both with increased risk and poor outcome of IA.^[15]^ By multivariable analysis among Aspergillus infection patients, antifungal therapy (HR 0.383, 95% CI 0.163–0.899, *P* = .027) was associated with improved survival, whereas accumulated dose of systemic steroids > 700 mg (HR 2.452, 95% CI 1.134–5.300, *P* = .023) and respiratory failure at admission (HR 5.983, 95% CI 2.487–14.397, *P* < .001) were independently associated with increased mortality.^[16]^

Glucocorticoids have potent, pleiotropic effects on the immune system that can predispose patients to developing life-threatening IA. The functional effects of prolonged high-dose glucocorticoids on alveolar macrophages are critical, as these immune cells are the primary lines of the innate cellular defense against inhaled *Aspergillus conidia*.^[17]^ Even low concentrations of glucocorticoids reduce healthy human neutrophil-mediated hyphal damage by 20% after as little as 10 minutes of exposure.

The patient was immunocompromised and had several predisposing factors for invasive pulmonary infections, including: advanced age, bronchiectasis, and steroids. Patients treated with steroids are frequently at a high risk of developing opportunistic infections, and because of his initial failure to use appropriate antibiotics, his disease gradually progressed. Antibacterial treatment was changed when *Nocardia* and Aspergillus were cultured from the BALF fluid. Subsequently, despite the longer treatment duration, the patient achieved excellent clinical and radiological recovery.

## 4. Conclusions

In conclusion, co-infection with *Nocardia* and *A fumigatus* remains uncommon but is associated with significant mortality in immunocompromised patients, and traditional pathogen-detecting methods, including serology and culture, usually target common pathogens with low sensitivity which are more difficult to diagnose and treat, resulting in higher mortality.^[18]^ Therefore, clinical doctors must be more proactive in diagnosis, and as an emerging microbial identification system,16SrRNA PCR and next-generation sequencing (NGS) have been used for the clinical diagnosis of a variety of infectious diseases.^[19,20]^ We predict that this opportunistic pathogen will be reported more readily in clinical specimens with improved pathogen diagnostic methods, as well as enhanced clinical awareness. Again, the key of this case reminds us that we should be aware of 2 or more co-infection can occur in immunocompromised patients, and also the traditional diagnostic assays such as culture, smear are still play a very useful role in the clinical diagnosis.

## Acknowledgments

We would like to thank the patient for their great help in this report.

## Author contributions

**Data curation:** Lei Wang, Hui Li, Yang Liu.

**Investigation:** Lei Wang, Hui Li.

**Methodology:** Lei Wang.

**Writing – review & editing:** Lei Wang, Hui Li.
